# The use of social media art challenges to encourage arts engagement for mental wellbeing in the general population

**DOI:** 10.3389/fpsyg.2023.1113280

**Published:** 2023-03-08

**Authors:** Michelle B. Kelly, Brid Phillips, Christina R. Davies

**Affiliations:** Centre for Arts, Mental Health and Wellbeing WA, School of Allied Health, The University of Western Australia, Perth, WA, Australia

**Keywords:** arts, mental health, social media, wellbeing, challenges

## Introduction

Social media is an essential component of health promotion, education and communication (Hefler et al., 2020). Social media is a cost-effective health promotion tool, with many benefits including targeted messaging, community education, and functionality to reach both the general population and target populations (Hefler et al., 2020). In Australia, popular forms of social media include Facebook, Instagram, Twitter, TikTok, YouTube, Snapchat, and LinkedIn [Australian Communications Media Authority (ACMA), 2020]. In 2022, 83% of the Australian population were active social media users (Statista, 2022a).

Engagement in the arts provides many positive health benefits (Davies et al., 2014) with strong evidence that recreational arts engagement for enjoyment, entertainment or as a hobby enhances mental wellbeing (Davies and Pescud, 2020). The main art forms include performing arts; visual arts, design and craft; community and cultural festivals; literature, and online, digital and electronic arts (Davies and Clift, 2022). The public may participate in each art form by engaging in events and activities. In recent years, this has included social media art challenges such as Inktober, Culture Dance, and @tussenkunstenquarantaine's photo challenge. Social media art challenges encourage participants to make or participate in the arts in response to a creative task, assignment or instruction and share the creation on social media. Social media art challenges include encouraging people to learn a new skill and engage with an online community (Li, 2021). Health professionals should consider the use of social media art challenges as a health promotion strategy conducive to arts participation to enhance community mental health and wellbeing. Via a selection of case studies, the purpose of this article is to provide insight into social media art challenges and the benefits to participants to assist health professionals, policy makers and researchers in their use of social media as a health promotion strategy. This is a novel avenue for encouraging community engagement in the arts for mental wellbeing at a time when globally, the prevalence of mental health issues are increasing (World Health Organization., 2022a).

## Arts engagement to promote mental wellbeing

A combination of mental health workforce shortages and the rising economic costs of treating mental illness has increased attention toward health promotion initiatives that encourage community mental wellbeing (All-Party Parliamentary Group on Arts Health and Wellbeing, 2017; Mental Health Commission., 2018). One such mental health promotion strategy is engagement in the arts (All-Party Parliamentary Group on Arts Health and Wellbeing, 2017). Research suggests that adults who take part in two or more hours per week of arts engagement report significantly better mental wellbeing than those who engage in no arts or lower levels of engagement (Davies et al., 2016). Some of the mental health benefits of arts engagement include feelings of happiness, joy, increased confidence, self-esteem, self-understanding, reduced stress, increased relaxation and social connection (Davies et al., 2016; All-Party Parliamentary Group on Arts Health and Wellbeing, 2017). Public health professionals may consider a social media art challenge as a health promotion strategy to increase engagement in the arts for mental health benefits.

## Social media to promote art challenges

Social media is any web-based communication channel dedicated to community-based input, interaction, networking, content-sharing and/or collaboration (Klassen et al., 2018). This includes platforms such as Facebook, YouTube, Snapchat, Instagram, or purpose-built, private discussion forums for ‘closed' groups (Klassen et al., 2018). In Australia, Facebook is the most widely and frequently used platform, with ~11.5 million users, which represents more than 40% of the Australian population (Statista, 2022c). Although social media is a relatively new form of health promotion communication, it is now an essential health promotion tool, complementary to ‘traditional methods' of communication and more cost-effective than traditional communication methods such as television or radio advertising (Rayward et al., 2019; Hefler et al., 2020). Social media is a wide-reaching channel (Rayward et al., 2019), with easily collectable metrics useful for evaluation (Vassallo et al., 2021), and campaign settings that can be adjusted to increase exposure to evidence-based health information (Tribe et al., 2021). Despite the many benefits, public health professionals must weigh up both the positive and the negative aspects, as there is ongoing debate surrounding the impact of social media on mental health and wellbeing (Glaser et al., 2018). For example, social media use can have unintended negative consequences such as cyberbullying, and social comparisons—when social media users make comparisons with others which may cause distress (Radovic et al., 2017; Liu et al., 2019). User experience can vary considerably based on a range of complex individual differences and behaviors such as intended use of social media (e.g., for entertainment or social connection), how it is used (e.g., active social communication or passive content consumption); developmental differences of users, existing offline social support, and whether the user has a pre-existing mental health condition such as depression (Radovic et al., 2017; Glaser et al., 2018; Liu et al., 2019). Despite this, it is recommended in the literature to encourage audiences to use social media to purposefully connect with friends, family, or other members of the community by engaging and creating content, to help reduce unintended negative consequences (Radovic et al., 2017).

While considering both the positive and negative outcomes of social media use, public health professionals should consider innovative digital interventions to reach their target audience. This includes engaging content that facilitates sustained and healthy habits such as social media art challenges. Participating in a social media art challenge and actively interacting with the online community (e.g., creating content, tagging, commenting), may help to facilitate social connection, social support, reduce loneliness, and improve psychological wellbeing (Liu et al., 2019).

### Visual art challenges on social media

As public health professionals and policy makers face the challenge of trying to encourage the general population to practice healthy habits, an innovative approach which has led to improved wellbeing includes the ‘digital daily practice' (Brewster and Cox, 2019), where participants sign up to a creative task or challenge with an online community who are doing the same task or challenge. An example of a social media visual arts challenge that highlights this idea is ‘Inktober'; Inktober is a daily drawing task based on a published daily word list for example ‘snack', ‘kind', ‘trip', and ‘farm'. Inktober is designed to help people improve their drawing skills, network with other artists (professional or hobby), and grow a following for their work (Parker, 2014). Participants use ink, paper, and other art materials each day in October and publish a digital photo of their work on social media with the hashtag ‘#inktober'. The challenge includes simple guidelines, engagement with established artist mentors and video tutorials to encourage people to participate and strengthen their drawing skills. The hashtag (#), account tagging, resharing, the ‘like' features of social media and the Inktober social media account connect participants with the broader community. Participants can access advice via stories and reels, live drawing sessions, and inspirational photos. In 2022, the ‘Inktober' Instagram page had 1 million followers and the hashtag #inktober was used on 24.5 million posts (Parker, 2014). Since research suggests those who engage in the arts for two or more hours per week experience significantly better mental wellbeing (than none or lower levels of engagement) (Davies et al., 2016), social media art challenges such as Inktober which encourage daily participation over a one-month period can help to establish creative habits which may also enhance mental wellbeing.

### Dance challenges on social media

Globally, physical inactivity is a major public health concern (World Health Organization., 2022b). Studies suggest dancing offers many health benefits that can enhance emotional, physical, and spiritual wellbeing (Murcia et al., 2010). There is increasing evidence that dance can meet health and wellbeing needs through the unique combination of social inclusion, social connection, physical activity and creative expression (Collard-Stokes and Irons, 2022). Public health professionals should consider a social media dance challenge when trying to increase general population or a target group's mental wellbeing and physical activity (Collard-Stokes and Irons, 2022), since internet-delivered programs have broad reach and can produce positive changes to physical health (Rayward et al., 2019).

TikTok is popular amongst young audiences. In January 2023, 21.5% of its global audience were women age 18–24 years old, while male users in the same aged group made up 17% (Statista., 2023). Numerous TikTok dance challenges emerged during the COVID-19 pandemic (Warburton, 2022). For example, the ‘Culture Dance' TikTok challenge involves individuals sharing short videos of themselves first dancing to music in ‘everyday' clothing, followed by footage that switches to an outfit representing their cultural heritage or national dress (Warburton, 2022). In 2022, “Culture Dance” reached over 15 million people (Warburton, 2022). The possibility of becoming a ‘creator' via TikTok dance challenges invites everyday people to share a new idea, post a new dance routine, offer suggestions, and encourages others (Warburton, 2022). As TikTok is an ideal platform to reach large audiences (Comp et al., 2020) TikTok dance challenges could be an effective way for health professionals to promote both mental wellbeing and physical activity to the general population and specific target groups.

### Photography challenges on social media

There is an increasing body of evidence that suggests photography enhances wellbeing (Brewster and Cox, 2019; Breckon and Lake, 2022). For example, the daily practice of creating a ‘photo-a-day' was found to improve participant reciprocity and empathy, facilitate social relationships, and encourage participants to engage in the real-world (Brewster and Cox, 2019). A randomized control trial of participants who took photographs of things that made them feel positive and hopeful over a 2-week period during the COVID-19 pandemic found that, after adjusting for baseline wellbeing, both wellbeing and post-traumatic growth were significantly higher in the intervention group compared to the control group, with this effect still apparent at 1-month follow-up (Read et al., 2022).

A photography arts challenge which emerged during the COVID-19 pandemic included those by leading art galleries including the Getty Museum and the Rijkskmuseum (Kapsová and Spálová, 2022). For example, the photography challenge @tussenkunstenquarantaine on Instagram, which translates to “between Arts and Quarantine” (Dedema and Fichman, 2021), where the general public were invited to choose an artwork from the gallery, use three household items to recreate the artwork in their home, and publish a photo of their art reproduction to their Instagram account with the tag ‘@tussenkunstenquarantaine' (Officier, 2020). The @tussenkunstenquarantaine challenge resulted in more than 200,000 followers and active participation from all around the world in just a few weeks of the challenge being created (Kapsová and Spálová, 2022), leading to far greater reach and impact than if the activity had been conducted face-to-face in the gallery or at school (Kapsová and Spálová, 2022).

## Conclusion

Innovative strategies are needed to contribute to community mental wellbeing at a time when mental health conditions are widespread, undertreated and insufficiently resourced (World Health Organization., 2022a). While there is evidence that arts engagement positively impacts mental wellbeing (Davies and Pescud, 2020), there is limited information about arts-health promotion strategies, especially social media art challenges. Because of the cost effectiveness, interactivity, accessible metrics, and wide-reaching audiences (Statista, 2022b), and opportunities to increase exposure to evidence-based health information and messages, social media art challenges which encourage creativity and community interaction, such as Inktober, @tussenkunstenquarantaine, and Culture Dance, should be considered by health professionals, policy makers and researchers as a method for promoting engagement in the arts for mental wellbeing, enjoyment, and social inclusion to the general public and specific target groups.

## Author contributions

MK and CD conceived the idea. MK, CD, and BP contributed to the writing, critical review, and final version of the opinion piece. All authors read and approved the final manuscript.
